# “FREED instils a bit of hope in the eating disorder community… that things can change.”: an investigation of clinician views on implementation facilitators and challenges from the rapid scaling of the First Episode Rapid Early Intervention for Eating Disorders programme

**DOI:** 10.3389/fpsyt.2024.1327328

**Published:** 2024-03-26

**Authors:** Lucy Hyam, Olivia Yeadon-Ray, Katie Richards, Amy Semple, Karina Allen, Jill Owens, Aileen Jackson, Laura Semple, Danielle Glennon, Giulia Di Clemente, Jess Griffiths, Regan Mills, Ulrike Schmidt

**Affiliations:** ^1^ Centre for Research in Eating and Weight Disorders, Department of Psychological Medicine, Institute of Psychiatry, Psychology and Neuroscience, King’s College London, London, United Kingdom; ^2^ Department of Psychosis Studies, Institute of Psychiatry, Psychology and Neuroscience, King’s College London, London, United Kingdom; ^3^ Centre for Implementation Science, Health Service and Population Research Department, Institute of Psychiatry, Psychology and Neuroscience, King’s College London, London, United Kingdom; ^4^ Health Innovation Network, Academic Health Science Network, London, United Kingdom; ^5^ Eating Disorders Outpatient Service, South London and Maudsley National Health Service (NHS) Foundation Trust, London, United Kingdom; ^6^ The Academic Health Science Network, Hosted by Manchester Foundation Trust, London, United Kingdom

**Keywords:** feeding and eating disorders, national health services, early medical intervention, mental health services, emerging adulthood

## Abstract

**Introduction:**

First Episode Rapid Early Intervention for Eating Disorders (FREED) is the leading eating disorder (ED) early intervention model for young people. Research has shown that it reduces the duration of untreated illness, improves clinical outcomes, and has cost savings. However, less is known about the experience of implementing FREED. This study aimed to investigate the views and experiences of adopting, implementing, and sustaining FREED from the perspective of clinical staff.

**Methods:**

Seven focus groups were conducted involving 26 clinicians. Thematic analysis was used, with the Non-Adoption, Abandonment and Challenges to Scale-up, Spread and Sustainability (The NASSS framework) framework being applied to organise subthemes and determine facilitators and barriers. The NASSS framework was also used to rate the complexity of themes as either simple (straightforward, predictable, few components), complicated (multiple interrelating components), or complex (dynamic, unpredictable, not easily divisible into constituent components).

**Results:**

There were 16 subthemes identified under seven broader themes representing each domain of the NASSS framework. Key barriers and areas of complexity included factors related to EDs as an illness (e.g., high acuity and prevalence), and organisational complexity (e.g., staffing shortages, lack of managerial/team support). Key facilitators included positive clinician/adopter attitudes, a supportive national network, and the ability for FREED to be flexible/adaptable over time.

**Conclusion:**

The FREED model appears to be desirable to clinical staff. Wider team and managerial support was perceived to be particularly important to its successful implementation, as were the national network and supervision. Key areas of complexity include staffing issues and high ED acuity/prevalence. These barriers to implementation need to be managed and investment continued to expand and improve early intervention for EDs further.

## Introduction

Eating disorders (EDs) such as anorexia nervosa (AN), bulimia nervosa (BN), and binge eating disorder (BED) are severe, deadly, and highly disabling psychiatric illnesses (1). Intervening early is paramount to full recovery, but many EDs “go under the radar,” undetected and untreated (2). For young people with EDs, prolonged malnourishment and compensatory behaviours (e.g., purging) can have a profound impact on brain development as well as lasting physical implications, such as impaired bone development or dental erosion (3, 4). EDs are also highly associated with mental health comorbidities, increasing the burden of disease, and considerable impairments in quality of life (5, 6). The average duration of untreated illness is between 2.5 years for AN up to over 5 years for BED, demonstrating extensive delays to starting treatment (7).

The reasons for prolonged duration of untreated ED (DUED) are multifaceted. After overcoming individual barriers to seeking help, such as stigma and shame, those with EDs face service-related issues such as long waiting lists and diagnostic gatekeeping procedures (8–10). For example, in many healthcare systems across the globe, access to a specialist ED service is preceded by a primary healthcare professional (e.g., general practitioner). These professionals often have limited training on EDs and report a lack of confidence managing these cases (10). Individuals with EDs report that primary healthcare professionals lack sufficient understanding and knowledge of symptoms and therefore fail to make a timely diagnosis or referral (11). Furthermore, access to specialist treatment is often not equitable for all ED diagnoses; in some areas of the UK, only low weight ED diagnoses are seen by specialist National Health Service (NHS) ED services (8).

For patients, carers, and ED specialists, accessible evidence-based treatment and early intervention are key priorities for research (10, 12). However, there is little research on how to reduce DUED and research on early intervention in practice for EDs is growing but limited (13). Currently, the leading early intervention model for EDs is First Episode Rapid Early Intervention for Eating Disorders (FREED). FREED is a transdiagnostic service model and care pathway for patients aged 16–25 with a recent onset ED (≤3-year duration). Young people within this age group, “emerging adults,” face unique challenges such as changing support networks, multiple life transitions, and growing independence from the family (14). Accordingly, FREED is modelled on early intervention approaches for first episode psychosis to provide rapid and personalised care tailored to this developmental and early illness stage, with core aims to reduce DUED and improve treatment outcomes (15). It does this by providing rapid access to ED assessment and treatment and by adapting existing evidence-based ED treatments to the age and developmental stage of emerging adults with a recent-onset ED. FREED care package adaptations include proactive reinforcement of help-seeking, a greater emphasis than usual on making early dietary change, attention to social media use, transitions and emerging adulthood, and an increased focus on family or close other involvement. For a full description of how FREED differs from conventional ED treatment, see Fukutomi et al. (16).

**Figure 1:**
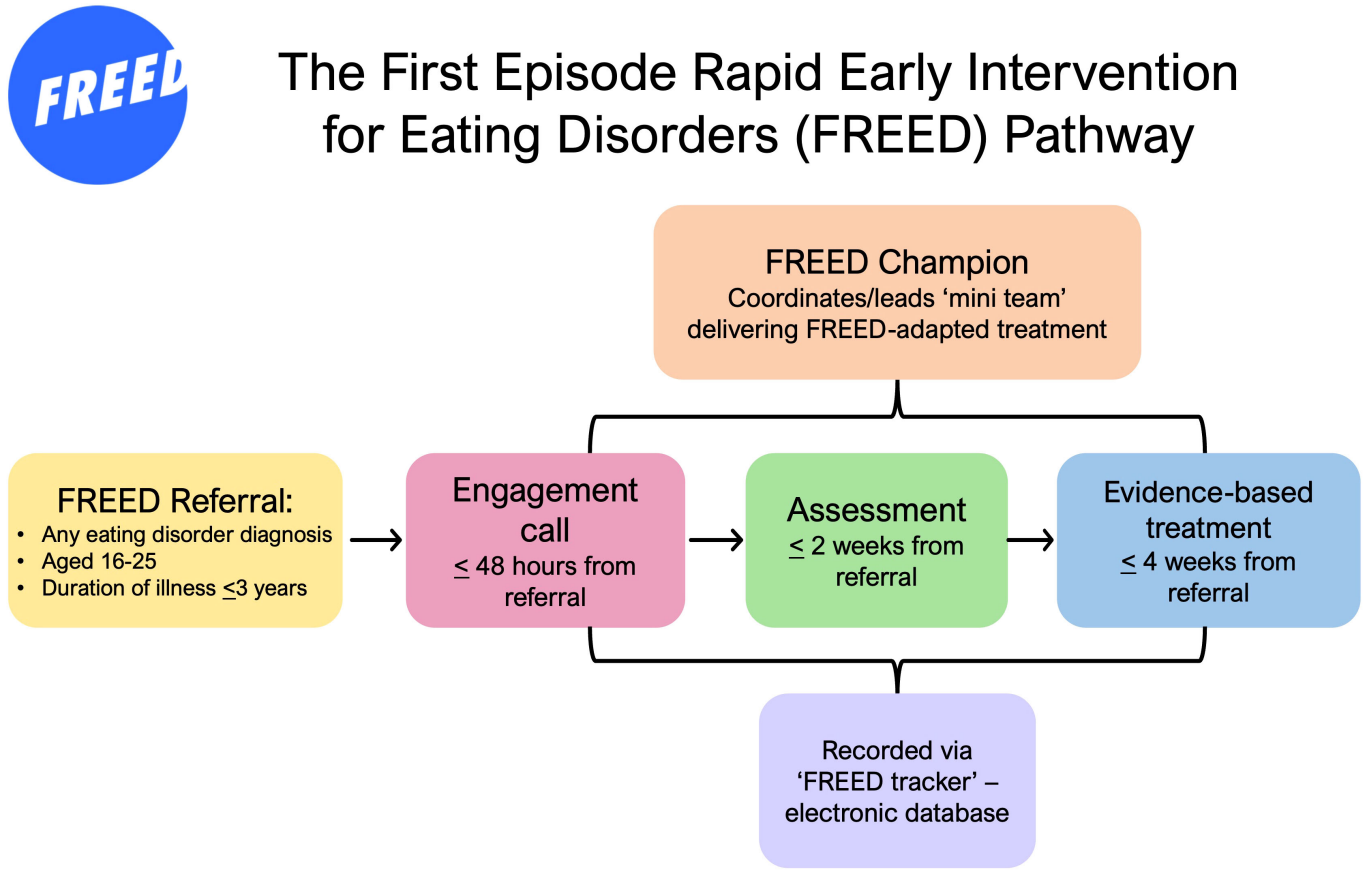
The FREED pathway.

FREED operates as a service within a service. Typically, a FREED Champion is appointed to manage patient throughput and oversee the provision of FREED and a FREED “mini-team” assist in delivering youth-friendly assessments and treatments. Active patient engagement and outreach is encouraged via a telephone call within 48 h of referral. Waiting time targets are 2 weeks from referral to assessment and 4 weeks from referral to starting evidence-based treatment to facilitate early recovery and minimise the likelihood of illness progression (17) All FREED services collect deidentified data on patient referrals and treatment outcomes via an electronic spreadsheet “tracker.” The FREED pathway and processes are demonstrated in **Figure 1**.

FREED was initially evaluated in single-site and multisite studies, where it was shown to lead to reductions in DUED, improve treatment uptake, and reduce waiting times for assessment and treatment (17). FREED also demonstrated notable cost-savings (18) and improvements in clinical outcomes (11, 13). In light of these findings, attempts were made to increase the provision of FREED further across the UK (15). The spread and scaleup of innovative service models and care pathways across healthcare systems is notoriously difficult, and even when innovations are evidence-based, they rarely achieve extensive uptake (19). When innovations or settings are complex, they are less likely to be adopted, scaled-up, spread, and sustained (20). The NHS is particularly slow at adopting new healthcare technologies and innovations (21). As the largest single unified healthcare system in the world, it is a complex system (22), i.e., characterised by uncertainty and unpredictability (19). Specifically, it is fraught with challenges, such as limited budgets, increasing demand and pressure, and difficulties in creating a culture for innovation (22). Given the lack of implementation research in the ED field (23), it is imperative to evaluate endeavours in this area to bridge research to real-world application.

In more recent years, key organisations have supported implementation projects across the NHS (22, 24). In April 2020, the Health Innovation Network (formally the Academic Health Science Network) supported the rapid scaling of FREED across England, providing national programme management and regional implementation support to ED services. This scaling of FREED took place between 2020 and 2023, largely during the COVID-19 pandemic. Initial quantitative evidence suggests that the beneficial effects of FREED are replicating across the country (25). However, there is a need to understand the views and experiences of those involved in implementing FREED during national scaling. A qualitative evaluation of regional programme leads involved in scaling FREED across England has been completed, revealing widespread positive attitudes and support for the pathway and for early intervention, from clinicians to stakeholder groups. However, implementation was noted to be fragile in some areas, and fidelity to the model variable across the country. Prominent capacity issues (e.g., unfilled posts, high staff turnover) were also considered a threat to the sustainability of early intervention (26).

Investigations into clinician attitudes are also important to the evaluation of FREED. Clinician views and appraisals of an innovation are critical to successful and sustained implementation (27). A qualitative analysis involving early adopters of FREED (adopted before 2021) showed positive and enthusiastic clinician attitudes toward FREED as key drivers for implementation, and FREED was seen to be compatible and adaptable to services (28). Clinicians also highlighted scepticism about the ability to implement FREED due to limited resources and concern about the impact on non-FREED patients. Early adopters of an innovation are argued to differ from later adopters, who are understudied in implementation science and are more likely to have limited resources and competing priorities (29). Thus, continuing to investigate clinician views in the later stages of implementation and national scaling will further elucidate the feasibility and acceptability of implementing an early intervention service in the NHS. This will also inform future planning for FREED and give insight into the sustainability and evolution of FREED over time.

A framework for understanding the multiple interacting domains of a complex system and how they affect a given innovation project is the Non-Adoption, Abandonment and Challenges to Scale-up, Spread and Sustainability (NASSS) framework (30). The NASSS framework focuses on the implementation of technology in healthcare. Here, we define FREED as the technology in question. The NASSS framework allows for the exploration of the multiple possible areas of complexity associated with implementing FREED in the “real world” by summarising seven domains where possible complexity may arise. These include the health condition that the technology (FREED) addresses (EDs); the features of the technology itself; the value proposition of FREED to the whole healthcare system (patients, carers, staff, etc.); the usability and acceptability of FREED for adopters (NHS ED service staff); organisational (NHS ED services) factors; wider context issues (e.g., policy-related drivers); and finally, the development and modification of FREED over time. Exploring each of these sources of complexity and the perceived facilitators and barriers to implementation will support efforts to sustain the adoption of FREED in services and inform work to improve local implementation fidelity.

The aims of this study are, first, to investigate the perceived challenges and facilitators to implementing FREED in ED services across England and adaptations made to the model during implementation, and second, to explore views on the sustainability of FREED, via the views of NHS ED service clinicians.

## Methods

### Design and materials

Ethical approval for this study was granted by King’s College London Ethics (MRA-21/22-26307). This study used a qualitative, exploratory design adopting a critical realist theoretical perspective. This view emphasises that, while an objective world exists, which can be understood through scientific endeavour, our learning and understanding is mediated through theoretical and research interpretation (31, 32). Accordingly, the NASSS framework was used to inform our understanding of the subjective experiences of clinical staff involved in implementing FREED.

The NASSS framework and NASSS complexity assessment tool (33) were used to inspire a focus group topic guide (**Supplementary Materials 1**) consisting of open-ended questions. These tools are designed to help users understand the interacting elements of an innovation project and areas where complexity can be reduced. LH drafted the topic guide, which was then refined in meetings with four other authors. The topic guide explored three broad areas with questions aiming to address each of the seven domains of the NASSS framework. The first section focused on getting FREED started, to investigate first impressions and the early stages of adopting FREED. The second focused on embedding FREED, to investigate the experience of running an early intervention service, and the challenges and facilitators experienced when implementing FREED. The third and final sections examined the wider context and future of FREED , to explore future developments or implementation sustainability. The topic guide was used flexibly to pursue and prioritise participants’ own thoughts and lines of discussion.

### Participants

FREED services become part of the FREED Network, overseen by a FREED National team, comprising clinicians and researchers from the South London and Maudsley NHS Foundation Trust, and King’s College London (KCL). Participants were purposefully sampled from FREED Network implementation supervision groups, which are virtual monthly support sessions organised for FREED Champions by the FREED National team. A total of 43 clinicians were emailed and informed that one of their upcoming implementation supervision groups would be replaced by the focus group session and to email the researchers if they were interested in participating. A total of 26 clinical staff agreed to take part who were mostly FREED Champions, but some were supporting the pathway in another way, e.g., as part of the FREED mini-team. Most participants were clinical/counselling psychologists or senior nurse practitioners, but professions also included occupational health therapists and assistant psychologists. The sample included clinicians from FREED services at different stages of implementation and from various areas of the country: London (*n* = 4), East of England (*n* = 3), Midlands (*n* = 7), North East and Yorkshire (*n* = 4), North West (*n* = 4), South West (*n* = 3), and South East (*n* = 1).

### Procedure

The focus groups were facilitated by two researchers, a researcher within the FREED National team based at KCL (LH, who was known to participants beforehand) and a Master’s student external to the FREED team based at KCL (OY). We thought that conducting focus groups during clinicians’ usual supervision meetings would increase the possibility to participate, compared with individual interviews, due to limited availability of staff. Additionally, these groups were seen as a comfortable, familiar space for clinicians to engage in meaningful reflection on implementation journeys. Individual interviews were conducted in a sample of early adopters of FREED (28), so it was felt that focus groups could complement these and possibly shed new and different findings from a more diverse group of participants. Focus groups are particularly useful for areas where little data exist (as in early intervention for EDs) (34). It was not expected that any power dynamics would substantially affect the results as the groups were led by junior researchers. While LH was known to participants beforehand, the supervision sessions that the focus groups took place in are encouraged to be a space for honest sharing and reflection, and we encouraged participants to share challenges and negative views as well as positive views.

Those who expressed interest were sent the information sheet and an electronic consent form to sign. Once all participants had joined the session, they were introduced and reminded about the study aims. They were informed that their data could not be withdrawn after participating in the focus group but omissions from the transcript could be made on request. Participants were also informed that due to the nature of the focus group, other members may recognise them but the research team would not disclose participation to anyone else, also that transcriptions would be anonymised and any reference to names, places, or any other identifying information would be removed. The sessions were led by OY with support, prompting, and introductions from LH. As the groups took place via Microsoft Teams, the inbuilt recording function was used to video record the session. The focus group recordings and automatic transcripts were then downloaded from Microsoft Stream, transcribed verbatim by OY, checked and validated by LH, and then analysed. Participants did not review the transcripts or provide feedback on findings.

### Analysis

Data analysis was supported by NVivo 12 (QSR International Pty Ltd., 2020). Thematic analysis was used as this approach is well suited to the use of both inductive (data-driven) and deductive (theory-driven) processes (35, 36). Transcripts were first read through for data immersion by LH and OY. Then, inductive codes were generated from emerging notes from the data separately by LH and OY. These codes were iteratively reviewed during analysis of the transcripts to ensure all data were coded appropriately and cohesively. Then, codes were converged by LH with review from US and KA and defined into subthemes organised under seven broader themes aligning to the domains of the NASSS framework. Researcher disagreement on the converging of codes was minimal and resolved through discussion at meetings. Each subtheme was defined as a facilitator or barrier by LH with review from US and KA. The complexity of each domain was then rated as either simple (straightforward, predictable, few components), complicated (multiple interrelating components), or complex (dynamic, unpredictable, not easily divisible into constituent components) (20).

## Results

Seven virtual focus groups took place between April and May 2022. Group sizes ranged between two to six participants, and the average duration of the groups was 52.05 min (range 45.59 min–59.45 min). There were 16 subthemes identified under seven broader themes representing the domains of the NASSS framework (the condition [EDs], the technology [FREED], the value proposition, the adopters, the organisation, wider context, and emergence over time). Four subthemes were identified as barriers, six as facilitators, and six as both a facilitator and barrier. All themes, subthemes (and their rating as a facilitator, barrier, or both), and example quotations are included in **Table 1**.

**Table 1:** Summary of themes and example data.

Theme	Complexity	Subtheme	Example data	Facilitator or barrier
**The condition**	Complex	Eating disorder complexity and prevalence	“Patients coming to the service have obviously increased… there’s a lot… more and a lot sicker clients.” (P69)	Barrier
**Technology**	Complicated	FREED National team support and resources	I think the materials are really good, the psychoeducation materials… we use those a lot with our clients and even people who may be in that age bracket but not on the FREED pathway. I think the social media is huge for a lot of the people that we see.” (P36)	Facilitator
		Fit between FREED and ED service	“…In a way, that FREED patient group have been slightly different to what we’re usually used to working with. So… a few… bits like that we’ve had to alter and think about. (P54)	Facilitator
		Partial or step-wise implementation of FREED	“We are an adult service so we don’t do the, 16 bit, we only do 18 to 25.” (P21)	Barrier
		Early intervention in practice	“When they clearly don’t want to engage with you, because it’s a real fine clinical decision that you make there… At what point does it become… chasing the patient too much.” (P44)	Facilitator and barrier
**Value proposition**	Complicated	Clear patient value and benefit	“I’ve learned things about like diet TikTok… that I never even would have thought of… I think when you… talk about it with young people, they can really get on board… with that.” (P16)	Facilitator
		Desire to increase efficacy and reach of early intervention	“The whole team was… noticing how beneficial it’s been, and we’re trying to get more funding at the moment then to expand to the other groups. (P54)	Facilitator and barrier
		Not meeting treatment waiting times	“We’re assessing people quickly and we’re offering other things like workshops and wellbeing calls with our support worker, but they’re not getting into intervention… within the four weeks that we’d hope.” (P12)	Barrier
**Adopters**	Simple	The FREED Champion	“I think having that regular training of what FREED is and reminding people about what it is has helped to embed it.” (P25)	Facilitator and barrier
		Excited, but trepidatious	“I think everybody was excited about the start of FREED and thinking that we could get people seen quicker, but we were unsure of numbers.” (P58)	Facilitator
**Organisation**	Complex	Staffing challenges	“In terms of like obstacles, I think our main one is, around resources, staffing issues.” (P65)	Barrier
		Wider team and management support	“Our managers, service leaders… is… really passionate about it as well. So, I think we’re in a really good position in that sense that everybody wants it to work.” (P31)	Facilitator and barrier
**Wider context**	Complicated	Impact of COVID-19	“It’s been still the ongoing impacts of staff absence as well, due to COVID which… we’re exposed to both inpatients and in the community… it’s still pretty harsh at times…” (P49)	Facilitator and barrier
		The FREED Network and Health Innovation Network support	“Those implementation meetings are so valuable… being able to share ideas and everybody’s… at various stages but it feels really supportive… like there’s always good ideas that come out of those meetings.” (P22)	Facilitator
		Increasing knowledge and connections with other organisations	“… The liaison that we’ve done… me and [colleague] are meeting with GPs next month… to talk about FREED… So, I think all that’s been, pretty good in terms of actually getting out there and doing it.” (P57)	Facilitator and barrier
**Emergence over time**	Simple	The future of FREED	“How do we implement it and continue to implement it in a way that it was designed to be. And what are, appropriate or satisfactory, kind of, alterations to it?” (P23)	Facilitator

FREED, First Episode Rapid Early Intervention for Eating Disorders; ED, Eating disorder; GPs, General Practitioners.

### Theme 1: the condition (EDs)

#### Eating disorder complexity and prevalence

Participants often discussed the complex nature of EDs and treating them. FREED patients were perceived to sometimes show “*high levels of ambivalence*” (P12) about seeking help and were not always ready to make changes. However, some clinicians felt that FREED patients were able to make changes quickly and had “*a quick turnaround*” (P63).

“*I think there is something different about the FREED population where people are much more… flexible in their thinking and… can make changes quickly. That's not to say you can't make changes quickly if you've had an eating disorder for a long time, but you definitely notice… with a proportion of those clients coming through… that's a really different experience for you as a clinician*.” (P22)

Participants felt that since the COVID-19 pandemic, there was a “*peak in acuity*” (P12). Clinicians described seeing more patients with a “*mixed presentation, often with comorbidity*” (P25), who were “*a lot sicker*” (P69), becoming “*very unwell…very quickly*” (P31). The increasing complexity of cases was challenging to balance while implementing FREED, as these cases often required urgent prioritisation and a longer course of treatment.

### Theme 2: the technology (FREED)

#### FREED National team support and resources

Participants often cited the FREED National team (South London and Maudsley NHS Foundation Trust and King’s College London) as a key facilitator to implementing FREED. The team were described as “*friendly*” (P25), “*welcoming*” (P25), and “*supportive… the whole way through*” (P57). Implementation support was consistently described as “*helpful*” (P12, P16, P17, P21, P25), and clinicians felt that there were “*no stupid questions*” (P16).

The online training package and resources were highlighted as “*really good*” (P58) and “*useful*” (P21, P23) for clinicians and for new starters in the service. Having FREED resources and psychoeducational material freely available meant that clinicians did not have to spend time creating new materials tailored to the FREED ethos, and it was felt that these were well-suited to patients’ needs. FREED resources were often used more widely than just for FREED patients:

“*…I think the materials are really good, the psychoeducation materials… we use those quite… a lot with our clients and even people who may be in that age bracket but not on the FREED pathway… I think the social media is huge for a lot of the people that we see, so that one’s been particularly… helpful.*” (P36)

Three participants (P44, P47, P68) suggested creating a directory or online group with everyone in the FREED Network (i.e., FREED Champions across the country, FREED National team contacts) so that Champions and clinicians could contact each other for advice/resource sharing.

#### Fit between FREED and ED service

The pathway was generally described as “*fitting alongside the service*” (P68), “*very flexible*” (P22), and as “*more of an adjustment*” (P25) rather than a whole service change. Some participants discussed how more work was required depending on “*what kind of service you're trying to launch FREED into*” (P12). For example, in some cases implementing FREED required adjusting administrative processes (e.g., rethinking how patients are allocated to therapists, or creating a new referral route for FREED patients). In some cases, this was “*a bit of a challenge*” (P49).

When discussing how FREED was different to standard practice, one participant reflected on FREED assessments:

“*I feel like FREED assessment is… very different to what anyone else does in the service. It is motivational… getting the clients on board…to get them to change, like…setting that goal at the end of assessment. I think it's incredibly powerful to give people information, from, the get go*.” (P69)

Also, seeing patients rapidly and “*getting in contact… so early*” (56) was sometimes described as new for services, and, for adult services, seeing patients so soon after turning 18:

“*…it's kind of new for a lot of our clinicians to start working with someone immediately, who's just presented to services for the first time… particularly young people… So, we're working with someone who's only just turned 18. That's quite new for our team… we had extra training… from a Child and Adolescent Mental Health Services (CAMHS) practitioner, just giving us some more tools and techniques what it's like working with, adolescents*.” (P54)

#### Partial or stepwise implementation of FREED

Some services changed the patient groups accepted under FREED, “*adapting FREED for what local provisions…*” (P51) are available. Some services “*lacked funding*” (P54) to offer FREED to the whole population, often because of how their services were commissioned (commissioning in the NHS refers to how healthcare services and resources are planned to meet the needs of the population, including where funding is allocated). For example, there were descriptions of FREED only being made available to parts of the catchment area covered by the service (P12, P21). However, there were often plans to eventually expand FREED provision.

“*We're only covering one patch…of our normal location…we're meant to be expanding, but…we're not quite there yet*.” (P12).

Another alteration described was making FREED available for a reduced age range (P25, P47).

“*…even though we are an all-age service, we're only offering it to 18-25…with the…very clear plan that we will be…opening it up to 16, 17-year-olds*.” (P25)

FREED is designed to treat all recent-onset EDs. In some cases, FREED was only made available for certain diagnoses or ED “severity” levels. The Diagnostic and Statistical Manual for Mental Disorders (fifth edition) categorises markers to dimensionally measure the severity of EDs. For AN, severity is measured by body mass index (BMI; kg/m^2^); for BN, severity is measured by frequency of inappropriate compensatory behaviours (e.g., vomiting), and for BED, severity is measured by frequency of binge eating episodes.

“*…our service is only commissioned for…anorexia…and bulimia…so…we're not commissioned at all for any work with binge eating disorder or…any of the other eating disorders.” (P31); “we've had…take the…mild to moderate…within FREED, but not severe because…there is just me…in the service*.” (P68)

#### Early intervention in practice

Several participants mentioned challenges in navigating how early intervention principles work in practice. For example, having regular discussions within teams about the difference between “*early intervention and what's preventative*” (P16) and deciding “*what is the most appropriate treatment*” (P51), for example, which interventions are more appropriate for “milder” ED cases. Where FREED encourages treatment recommended by The National Institute for Health and Care Excellence (NICE) for all patients meeting ED diagnostic criteria, some clinicians wondered if shorter, less intensive interventions could be used that are not recognised in NICE guidelines but are “*still in line with the FREED principles…but…so that it's manageable if…it's being used in services where there is this…milder eating disorder presentation that's accepted.*” (P12)

FREED encourages clinicians to reach out to patients more than they typically would to try and boost early engagement and help-seeking, and clinicians discussed some challenges doing this in practice when patients “*don't want to engage*” (P44). Some clinicians discussed starting to use a FREED mobile phone to contact patients. In these cases, clinicians described the importance of creating “*boundaries*” (P45) when receiving responses out of hours (P23, P44, P45), but that in general it was “*so much easier*” (P21) to contact patients in this way (e.g., via WhatsApp).

Lastly, clinicians discussed difficulties understanding the concept of DUED. Within FREED, DUED is calculated by estimating the time between clinical onset of the ED and first contact with a specialist ED service. If this period is ≤3 years (for any ED diagnosis), the patient is included on the FREED pathway. DUED was perceived as “*quite… confusing… at the start*” (P17), and some teams “*got really stuck on… the calculation of the DUED*” (P22). Participants also felt that some “*leeway*” (P44) on DUED criteria was desirable, to allow more patients to be treated under the FREED pathway, especially in cases where patients delayed contacting services because of the COVID-19 pandemic:

“*For some patients, that delay… for them referring themselves to us, meant that they were outside of… the DUED, for FREED*.” (P44)

### Theme 3: value proposition

#### Clear patient value and benefit

Participants frequently discussed that working with FREED patients and being able to offer rapid intervention was encouraging. Participants saw patients making “*changes quickly*” (P22) in behaviours and symptoms and felt that early intervention had prevented patients “*on the verge of a hospital admission*” (P36) from deteriorating. While many participants discussed that due to staff resource, they could not always see patients or start treatment within the recommended time frames, when they did, “*it works really well*” (P51) and is “*really fulfilling…and really positive*” (P64). Participants described that, for patients, they “*didn't think…they would be able to get help that quickly*” (P21).

“*Because of the quick turnaround, when these people were severely unwell presenting for the first time, because there wasn't a…long waiting list…, we were able to act upon it…So that's really reassuring that FREED has a place which is vital to keep our patients safe…with the clients I've seen on my caseload*, ***seeing them really bloom into who they wanted to be*** *. Really looking at, achieving those goals such as going to university or moving in with their partner or living independently or whatever it might be, those big life events that fall within that sort of 18 to 25 category…so, I suppose that evidence that I've seen from the patients is that it's been very, very beneficial*.” (P63)

The engagement call was described as “*almost revolutionary*” (P68). Participants felt that patients were “*surprised*” (P21) and “*impressed*” (P47) and found it “*amazing*” (P23) to be contacted so quickly, and that “*people are so grateful for that, that initial call*” (P58). In addition, the engagement call was felt to ease “*really nervous*” (P68) patients. One clinician reflected that for one patient: “*I don't think she would have come to the assessment without that engagement call*.” (P58) Another benefit to the call included being able to “*get so much better information than the GP [general practitioner] often passes on…and it helps us to think about what the most appropriate thing to do next is.”* (P58).

Overall, the engagement call was viewed as “*great for the person’s experience as well as for us as a service*” (P31). Various services had started to adopt these for all patients in the wider service, not just potential FREED patients (P31, P57, P58).

When patient engagement was variable and there were “*small pockets of people*” (P31) who did not engage, participants reflected that “*FREED does its best…to try to work with that and explore that with people…which is really good*.” (P36) Furthermore, it was felt that, because of FREED, services had “*spent more time engaging in patients generally than maybe we would of before…just getting to know them.”* (P45) One participant mentioned that they felt it important for the future to investigate patient engagement and trying to improve this within FREED (P51).

#### Desire to increase effectiveness and reach of early intervention

Some clinicians expressed concerns about whether non-FREED patients have to “*wait longer*” (P25) for treatment, and “*not being able to offer [FREED] to some people… can be frustrating*” (P23). However, participants also acknowledged that expanding the provision of an early intervention service would be tricky:

“*I think it would be nice if… FREED was available to everyone of every age at some point… but that's obviously a much bigger, issue… you'd have to have so many more staff implement that*.” (P21)

One participant discussed whether groups who are typically under-served in ED services, such as black and minority ethnic groups, have equitable access to FREED and whether more research could be done on accessibility (P22). Another participant described how their service had started to “*collect additional data on protected characteristics*” (P25) as part of FREED to investigate service accessibility.

In addition, participants described how, even though FREED was designed to reduce service-related barriers to ED treatment, there were still issues around access. For example, patients who moved between their university and home localities needed to be reviewed and transferred by primary care services (e.g., general practitioners [“GPs”]) before transitioning between FREED services. These patients could sometimes “*slip through [the] net*” (P44). Similarly, transitions between child and adolescent to adult ED services were sometimes “*difficult*” (P52) to navigate within a FREED service.

“*You know, we've got… a huge… student population… and sometimes it is really tricky to get people into other services because the referral needs to come from a GP…*” (P36)

Participants felt that there was an opportunity to use FREED to make these kinds of transitions better for patients.

“*… you know, if we can, use the FREED model in relation to… holding patients until they're engaged with their university FREED champion*.” (P44).

#### Not meeting treatment waiting times

Six participants discussed the “*challenge*” (P21) of meeting the FREED waiting time targets for assessment and treatment and some clinicians felt that they were “*lagging*” (P31) or had “*fallen behind*” (P23). Not being able to achieve these targets was “*confusing people, particularly parents*” (P12) who expected quicker access to treatment under FREED.

“*…all services… [are] probably gonna be struggling at the minute, so you might not be able to meet those targets, but we're just doing the best that we can*.” (P25)

### Theme 4: adopters

#### The FREED Champion

Participants who were FREED Champions described a commitment to “*promoting FREED*” (P51) and “*reminding*” (P25) and “*refreshing*” (P12) the principles of FREED and supporting research to the wider team. Collecting data via the FREED tracker was a part of the role that some participants felt was “*frightening*” (54) and “*anxiety provoking… maybe unnecessarily*” (P44). One clinical lead reflected on the importance of the therapeutic relationship between the patient and the FREED Champion:

“*Some of the comments from the young people have been, on discharge… about, the relationship with the FREED champion… about how lovely you were*” (P44)

#### Excited, but trepidatious

Many participants described working with FREED as “*exciting*” (P12, P16, P25, P21, P22, P23, P58, P68). Participants were interested in the “*research and importance of early intervention*” (P25) and the potential to offer care for a “*wider presentation of people*” (P21) in “*a timely manner*” (P23) was exciting. At the same time, participants were “*trepidatious*” (P16), “*scared*” (P22) or found it “*overwhelming*” (P17) to think about how FREED would be implemented.

“*…practically, how is this gonna happen? It sounds fabulous but how will we make it work?*” (P21)

FREED was perceived as aligning with clinicians’ values and the way they liked to work. For example, FREED’s “*strong family component*” (P12), “*transition work*” (P12, P16), and “*social media aspect*” (P16) were noted as features of the model that aligned with clinicians’ own interests. It was recognised that family involvement is not usually considered the “*biggest priority*” (P49) in conventional adult ED treatment.

One participant within an adult service described that introducing FREED led to more learning within the team about the needs of young people, transitions, and the concept of emerging adulthood.

“*…being an adult service, we've had to…do more learning…of the particular needs of young people…things like the social media and…the engagement issues, issues around transitions. So*, ***we've learned as a team, really, and upskilled*** *…*” (P44)

### Theme 5: organisation

#### Staffing challenges

Participants described how they were not “*fully resourced*” (P47). This was described as a barrier to implementing FREED effectively as it impacted adherence to the FREED assessment and treatment waiting time targets. Particularly, there was a lack of staff to deliver NICE-concordant treatment; for example, one participant described: “*I’m the only person in the mini-team that can offer MANTRA*.” (P22). The Maudsley Model of Anorexia Nervosa Treatment for Adults (MANTRA) is a NICE recommended first-line therapy for adults with anorexia nervosa.

Another participant felt they simply did not “*have the numbers to… ensure we're hitting those targets*.” (P65).

“*I think staffing is predominantly quite a big factor… just in terms of having capacity to see people… within the time frame*.” (P23)

Some participants discussed that new staff were being recruited into the service, which they were “*optimistic*” (P65) about. However, they emphasised the need to train these staff to ensure that “*everyone's really aware of what FREED is… so that we make sure that… we are adhering to the model.”* (P25) In some cases where services attempted to recruit new staff, these posts were left unfilled due to nationwide NHS recruitment issues:

“*There'd been a post for a psychologist, no one… picked it up. Then they were trying to tweak it to make it more broader… I don't think that's even been put out again*.” (P17)

#### Wider team and management support

The most densely coded subtheme included participants’ descriptions of and experiences with management and their teams and the impact on FREED.

“*There has been a few people [patients] where they've been picked up really quickly and they've ran with it and needed a short piece of intervention and…* ***there's been a few kind of light bulb moments with the team of being like, oh yeah this is what FREED is. This is, why early intervention’s, great*.”** (P12)

Some teams expressed having a lot of support from their colleagues and management, that FREED was “*keenly adopted by the service*” (P23) and teams and managers were “*really passionate about it*” (P31) and “*dedicated to doing it*” (P21). Unsurprisingly, a supportive management and team were considered a huge facilitator to FREED implementation and made the “*biggest difference*” for participants (P58).

“*Everyone was keen to just muck-in and like, right, roll your sleeves up, get on with it, we'll see what happens. It might not be the perfect… launch that we planned and it might just be, learning as we go along, but… luckily everyone was enthusiastic about the model*.” (P12)

A supportive FREED huddle and “*dedicated… willing… and able*” (P31) mini-team were considered highly important. These structures were felt to keep “*it all together*” and were “*a place to capture… are we sticking to this… is there a fidelity to the model*” (P22).

There were some descriptions of hesitancy within the team about doing something new, or the potential of an increased workload: “*…a lot of the clinicians on the ground floor worry that it’s going to be additional work… that's nothing to do with FREED, that's change as a whole, isn't it.”* (P51)

In contrast, a lack of team and management support was perceived as a barrier to FREED implementation. Some participants felt that being the FREED Champion meant that they were the “*only person*” (P16, P17) supporting early intervention within the service, which was felt to cause “*its own issues*” (P16). Participants described being treated as a “*separate… self-serving*” (P12) team by management, which contrasts with the intention of having FREED run as a “service within a service.” This had a knock-on effect where clinicians felt that there was reluctance within the team to engage with FREED, for example, a “*reluctance… to, become overly familiar with FREED*” (P23) or “*pick up… FREED clients for therapy*” (P12).

Ultimately, participants sometimes felt that they needed to remind their teams/management that FREED was not “*something completely different*” (P25).

“*I’d think… our small team and our service are aware of… FREED, what it involves, what the criteria is, but not necessarily… higher management… knowing what FREED is and understanding… that we are a service within a service. We're not a separate entity… I think sometimes it gets confused and they're not quite actually sure what FREED is*.” (P17)

When facing resistance or misconceptions about FREED within the wider team, participants expressed feeling “*demoralized*” (P16). One participant particularly felt that, despite their efforts, FREED was not “*warmly accepted*” and “*neglected*,” leading them to feel “*frustrated*,” like they were “*fighting a losing battle*” (P63).

### Theme 6: wider context

#### Impact of COVID-19

The COVID-19 pandemic affected FREED in various ways. Two participants reflected that the pandemic “*delayed*” (P47) and “*scuppered*” (P12) their launch plans, as, for example, key staff were being redeployed. It also stopped plans to communicate FREED more widely, for example with primary care services, which may “*have helped to embed FREED better*” (P21).

The move to virtual working was perceived as both positive and negative for different reasons. Participants found this change “*difficult*” (P17) and felt it created a “*disconnect*” (P16) within the team, disrupting normal ways of communication and preventing “*conversations about what FREED is*” (P12). In contrast, participants reflected how virtual assessments/treatment helped with patient engagement, leading to “*less DNAs [did not attend]*” (P17). Even though services had restarted face-to-face appointments at the time of the focus groups, some participants reflected that the flexibility was suitable for FREED patients specifically and sometimes worked “*better*” (P23) than face-to-face contact:

“*Going to the virtual and… this kind of client group, I think works quite well… and we’re still doing a lot of virtual work with the FREED clients who want it. We're offering them face to face, but… we're finding a lot of them… are liking the virtual, it fits around their lives better… they can do it in like, the breaks at uni or they can go to uni and we can carry on seeing them and, there's a bit more of that continuity*.” (P21)

#### The FREED Network and Health Innovation Network support

Monthly implementation supervision sessions hosted by the FREED National team with other FREED services were perceived as a key facilitator by most participants. These sessions helped to normalise the challenges of implementing FREED, which gave “*reassurance*” (P45) to clinicians and helped them “*feel less alone with it, and less pressured*.” (P36) As such, these monthly sessions were seen as a key support system for FREED Champions and clinicians.

These sessions were also instrumental to “*share learning*” (P45) and resources: “*I mean, why rewrite the wheel if, someone else has already done it and is willing to share*?” (P47). Participants felt that “*there's always good ideas that come out of those meetings*.” (P22)

“*I'm from a nursing background and have at times felt, a little bit inferior because I'm not a therapist or psychologist. But actually, the FREED network and regional supervision that we have has been really helpful… and everybody's been really honest about what the struggles are. And there's been no judgement and just offering of support and resources…*” (P64)

The local support provided by the Health Innovation Network was also seen as “*absolutely so important*” (P25) and “*really useful*” (P21) for implementing FREED, especially for linking clinicians with key FREED staff or other FREED services, and for keeping services “*on track*” (P47) with targets.

In general, the FREED Network and community was seen in a positive light and was commonly highlighted by participants.

“*I just want to mention, about the team, and… positives, is… the community. The FREED community, all the Champions, all the lovely people I've met, throughout this whole thing, sharing resources, which then informs better practice for all of our service users. I think that's one of the most amazing things that I've got from this as well.* ***I've met so many, passionate, clinicians… that have really helped shape how we do things, sharing resources, sharing ideas…*** *And actually meeting people all over the UK… it's lovely, it's really nice to share the word and spread it I think*.” (P68)

#### Increasing knowledge and connections with other organisations

Participants often used FREED to increase connectivity and knowledge of EDs and early intervention with other organisations. This included working with university wellbeing teams (P56, P51, P65) to identify individuals who would be eligible for treatment under the FREED pathway. One participant shared how starting FREED gave them the idea of making a podcast on EDs:

“*In terms of that creativity with us, I don't think us and the team would have ever have thought of, developing or making podcasts before, starting FREED*.” (P70)

Participants felt that there was an expectation from primary care services, such as GPs or community mental health teams, that a FREED service would see “*anything to do with eating*” (P51) and not necessarily diagnosable EDs, leading to “*inappropriate referrals*” (P25). However, participants also felt that some patients were still slipping through the gaps and therefore it was highly important to improve GPs’ knowledge of EDs to ensure potential FREED patients are not missed or turned away.

“*Even like a year on we were getting, patients come through that said ‘Oh, my GP told me you wouldn't accept me and they'd never heard of FREED and, we've done… so much promotion to GP, but… I don't know where it goes… so that's been difficult*.” (P21)

### Theme 7: emergence over time

#### The future of FREED

Participants felt positive about the future and sustainability of FREED and did not make many suggestions for changing the model. One participant added that moving forward, there was a need to monitor fidelity to the model:

“*…how do we implement it and continue to implement it in a way that it was designed, to be. And what are, appropriate or satisfactory, kind of, alterations to it?*” (P23)

General considerations for the future included bettering the transition process for patients from child and adolescent to adult mental health services and increasing collaboration across the FREED Network.

“***I’m looking forward and it’s exciting. I think there’s a bit of hope. I think that’s what FREED kind of instils is a bit of hope in that eating disorder community. That things can change***.” (P69)

## Discussion

This study aimed to investigate the perceived barriers and facilitators to implementing FREED from the perspective of clinicians at various stages of implementation, and to seek views on the sustainability of FREED. Prominent facilitators to implementation included clinician enthusiasm for early intervention and the value of a supportive national network. Barriers included lacking supportive management/team support and staffing gaps. The NASSS framework was used to offer a diverse understanding of the multiple interacting sources of possible complexity associated with implementing FREED.

The condition domain was rated as complex. Participants discussed that a barrier to early intervention was patient ambivalence/low help-seeking. However, some participants felt that FREED boosted patient engagement and worked well to explore ambivalence to treatment. A recent systematic review and meta-analysis showed that higher levels of denial and a perceived inability of others to provide help were found to drive low-help seeking in people with EDs (37). To overcome this barrier to early intervention, Radunz et al. suggest the use of targeted psychoeducational materials and codesign with people with lived experience. We are currently feasibility testing the use of an online help-seeking tool for young people meeting criteria for an ED (FREED-Mobile). This tool features psychoeducation on EDs and aims to increase motivation to seek treatment (Gruyczuk et al., submitted). Also, many FREED services have begun using peer support workers and the FREED National team are currently developing a peer support programme. Finding other ways to improve or manage ED-related complexity is highly important moving forward, especially considering increased referrals and rising demand for ED services (9, 38).

The technology domain was rated as complicated. Clinicians felt that there were sufficient resources, training, and avenues of support from the FREED National team to facilitate implementation. However, the ease of implementation was perceived to vary according to service design and some participants described instances where FREED was implemented partially or incrementally only, and therefore not available for all presentations of EDs, all intended ages, or the whole area covered by the service. This partial implementation needs to be monitored to assess whether over time services manage to offer FREED as intended or deliver FREED in name only (“FRINO”). Assessing and improving implementation fidelity will become an increasingly important task now FREED has been scaled nationally and is starting to scale internationally.

The value domain was rated as complicated as despite clinicians’ enthusiasm about the benefits of FREED, they were concerned about not meeting FREED waiting time targets. Clinicians felt that early intervention and the FREED ethos aligned with their own values and largely agreed on the benefits of FREED for patients. Specifically, participants valued the work undertaken to engage patients (e.g., phone calls/texts) and seeing patients make changes quickly. Participants expressed a desire to see early intervention available to all patient groups (i.e., those over age 25 and with DUED >3 years). However, participants also recognised that this would require even larger investment into staffing. Some participants discussed how they are already using FREED resources and engagement activities with other patient groups, demonstrating that FREED principles can be extended beyond the target patient group. For many services, FREED thus represented a starting point for early intervention and offered the flexibility to expand according to capacity. Participants also mentioned how FREED prompted creativity (e.g., new interventions and engagement activities), led to wider learning about emerging adulthood, and increased collaboration across the separate adult and child/adolescent services. Therefore, services embraced early intervention beyond the FREED blueprint, figuring out how to “do” early intervention in their local context. However, Richards et al. (28) found that early adopters of FREED who removed the upper age limit tended to reinstate it due to capacity issues, or felt that those aged over 25 did not benefit from FREED in the same way.

The adopters domain was rated as simple due to clinicians’ positivity and excitement about FREED. FREED Champion participants revealed feeling responsible for the embedding of FREED in their services and talked about their commitment to training new staff and providing regular updates at team meetings. Engaged and enthusiastic champions have consistently been cited as important for positive implementation outcomes (39, 40), and this research shows that enthusiasm is also present in later adopters of FREED. Considering the added burden of the COVID-19 pandemic and increased demand on services, it is a testament to these clinicians’ desire to see early intervention for EDs become reality.

In addition to driven champions, engaged and supportive teams and management are crucial for implementation success (40, 41). Many participants identified supportive leaders and teams which they perceived to be a key facilitator to implementation. However, a handful of participants felt that FREED was treated as a separate entity and consequently felt demotivated. It is suggested that one champion alone may not be enough to bring about change—multiple “champions” within the service, including those in leadership positions, are more effective (40). Another key perceived barrier to FREED implementation was NHS-wide recruitment issues and staffing gaps. Despite many clinicians being well-supported to operate FREED, the organisation domain was rated as complex due to ongoing systemic staffing issues. This qualitative research from the perspective of clinicians adds to repeated calls for urgent investment into adult NHS ED services to meet rising demand (9).

The wider context domain was rated as complicated. Inter-organisational networking and knowledge sharing via the FREED Network was identified as a key facilitator to FREED implementation, and participants had also begun working with other organisations such as university wellbeing teams to increase awareness of FREED in their local area. Despite discussion about the impact of COVID-19 on patient referrals (38) and team-working, participants often identified how the change to virtual working had benefits for early intervention by increasing patient engagement, which is consistent with findings from our study on early adopters of FREED (28). Overall, the FREED Network appeared to be highly important to clinicians and should thus be carefully supported and continued moving forward.

Two key questions concerning the emergence of FREED over time include (1) the ability of FREED to adapt to changing contexts and (2) organisational resilience (30). FREED was described as fairly flexible, and clinicians have indeed shown resilience in implementing FREED throughout the height of the COVID-19 pandemic, evidenced by data demonstrating that FREED is replicating at scale (25). In general, participants were positive and optimistic about the future of FREED. As such, the emergence domain was rated as simple. However, areas of instability relevant to the future include funding instability for national FREED steering and unstable ED service leadership and staffing.

### Limitations

A limitation of this research is that, out of 43 invited clinicians, 26 participated in the study. The main reasons for non-participation were clinicians not being available for the arranged session or not replying to the request to participate. It is possible that the sample represents clinicians who were particularly engaged with FREED and who regularly attended the supervision meetings. Clinicians who did not attend the supervision groups regularly or who were time-stretched may not have been able to commit to participation, and it is possible that these clinicians may have had different views not represented here. In addition, the invited clinicians were predominantly FREED Champions. While the researchers emphasised the importance of discussing both challenges and facilitators to implementation, clinicians external to the FREED mini-team may have expressed different views on the impact of FREED implementation. A service-led qualitative evaluation organised independent to the FREED National steering team may provide such insights and explore the views of a wider range of staff.

The views and experiences represented here are limited to clinicians working within an English NHS ED service. While there are important learning outcomes to be made, there is still limited evidence on how FREED may operate outside of this context in diverse cultures and different healthcare systems. FREED is now being adapted across the globe. In order to strengthen the general evidence base for early intervention for EDs and improve the generalisability and international relevance of FREED, it will be crucial to explore the attitudes and experiences of those involved in adapting the model to these contexts. Furthermore, research is needed on whether FREED affects treatment accessibility for those typically under-served in ED services, a knowledge gap also highlighted in our qualitative evaluation of innovation experts involved in the national scaling of FREED (26).

## Conclusion

FREED represents a starting point for early intervention for EDs, being the first dedicated pathway scaled nationally in the UK. Both quantitative and qualitative evidence suggests its cost benefits, promising clinical outcomes, and its value to patients and clinicians (15, 17, 18, 25, 42). While this is positive, recent research highlights that ED services, especially adult ED services, are severely underfunded and face growing treatment waitlists (9) and that the prevalence of EDs and ED symptoms globally increased following the COVID-19 pandemic (38, 43, 44). Investment into ED services and research must therefore be further expanded to improve early intervention in the UK, and beyond (45, 46). These data align with and further explore themes from a companion study interviewing implementation experts supporting FREED national scaling (26), and with an earlier sample of clinicians exclusively from early adopter sites (28). Common themes across these studies include FREED’s alignment to clinician values, staffing issues as a key barrier to implementation, and a desire to offer timely intervention to all patients regardless of illness duration and age.

## Data availability statement

The datasets presented in this article are not readily available because the participants of this study did not give written consent for their data to be shared publicly. Requests to access the datasets should be directed to lucy.e.hyam@kcl.ac.uk.

## Ethics statement

The studies involving humans were approved by the College Research Ethics Committee, King’s College London. The studies were conducted in accordance with the local legislation and institutional requirements. The participants provided their written informed consent to participate in this study.

## Author contributions

LH: Conceptualization, Data curation, Formal analysis, Investigation, Methodology, Writing – original draft, Writing – review & editing. OY-R: Data curation, Formal analysis, Methodology, Writing – review & editing, Investigation. KR: Conceptualization, Methodology, Writing – review & editing. AS: Conceptualization, Writing – review & editing. KA: Supervision, Methodology, Writing – review & editing. JO: Writing – review & editing. AJ: Writing – review & editing. LS: Writing – review & editing. DG: Writing – review & editing. GD: Writing – review & editing. JG: Writing – review & editing. RM: Writing – review & editing. US: Conceptualization, Supervision, Methodology, Writing – review & editing.
