# Supplementary material for: “FREED instils a bit of hope in the eating disorder community… that things can change.”: an investigation of clinician views on implementation facilitators and challenges from the rapid scaling of the First Episode Rapid Early Intervention for Eating Disorders programme
Source: Front Psychiatry. 2024 Mar 26;15:1327328. doi: 10.3389/fpsyt.2024.1327328 (PMC11002146; doi:10.3389/fpsyt.2024.1327328)
Supplement: Supplementary file 1 [file DataSheet_1.docx]

**Supplementary materials:** FREED Focus Groups Topic Guide

**Opening up the discussion:** (5 minutes)

1. Briefly, what is your role within FREED and how long have you and your team been involved with FREED?

**Getting started with FREED (~15 mins):**

1. If you cast your mind back - what were your general impressions of FREED when it was first introduced to the service?
   1. *Prompts: What about the rest of your team/site?*
   2. *What hopes or concerns did you or other members of your team have about introducing FREED?*
2. **Were any changes made *to the FREED model* to be able to implement it (for example, the targets or diagnoses seen in the pathway)?**
   1. *Prompts:* what were these changes? What did you think of these changes?
3. **Did you have to make any changes in your service to allow FREED to be adopted?**
   1. *Prompt: For example, in terms of processes, procedures, roles and responsibilities in your team/service*
4. The FREED model upholds the value of early intervention for eating disorders. Early intervention means getting help and support as soon as possible for someone with an eating disorder. In the FREED model, this means reaching people in the first three years of illness in order to more easily reverse the changes that the eating disorder makes to the brain, body and behaviour. Do you feel that the values of early intervention and FREED (*E.g., championing early intervention, targeting the 16-25 age group, emphasising the reversibility of changes made by ED)* are supported/championed by people in your service?
   1. *Prompts: Do you feel early intervention is supported more widely by your senior management, within your Trust and the NHS overall? (technology)*

**Embedding FREED (~15 mins)**

1. **What are the main facilitators to running FREED in your service?**
   1. *Which resources, meetings, structures, people or attitudes in particular have helped with implementation or the general running of FREED?*
   2. *Think back to when you were first implementing FREED, what do you think went particularly smoothly or ran well?*
   3. *What didn’t go so smoothly?*
   4. *Was there any specific support, resources, or organisations/people that made setting up and implementing FREED easier?*
   5. *Was there anyone driving FREED forward within or outside of your team?*
   6. *Was it easy or difficult to integrate FREED or its values in your team?*
   7. *Has FREED changed your team’s organisational routines?*
2. **What obstacles or challenges are you facing with implementing FREED in your service?** *Prompts: Have there been any resource or staffing related, structural, people- or attitude-related issues that have affected FREED implementation – if so how?*
3. What parts of FREED feel easier to implement and what parts of FREED feel difficult to implement in your service (targets, engagement call, distributing psychoed materials)?
   1. Is FREED being implemented as intended in your team (e.g., are people using the care package)?
4. What kind of support do you and your team require from the Academic Health Science Networks (AHSNs) and the FREED Network? *Prompts: what do you do when you have questions about FREED?*
5. How do your patients generally engage with FREED? *Prompts: Are there patients who are easier or harder to engage with FREED? What responses have you had from patients/carers?*
6. What do you and your team think about the data collection and performance feedback (e.g., data summaries)?

**Wider context and the future of FREED (~15 mins)**

1. **What future developments, additions, or changes would you like to see to the FREED model and the implementation support provided to teams?**
2. Can you comment on the role of implementation supervision meetings – do you think changes need to be made to these moving forward?
3. Did the COVID-19 pandemic affect your FREED pathway? How? *Prompts: What aspects of FREED (for example, the targets, patient groups you offer FREED to) have you had to change in your service in response to the pandemic?*
4. Do you think the FREED model and its targets are sustainable as we (attempt to) come out of the pandemic? *Prompts: Do you think FREED can adapt to and survive future clinical changes?*

**Closing (5-10 mins):**

1. Is there anything we have missed that you would like to discuss?
2. Of all the things we discussed, what to you is the most important?
